# Secondary spontaneous pneumothorax as an initial manifestation of lung cancer: a case report

**DOI:** 10.3389/fonc.2026.1832647

**Published:** 2026-06-08

**Authors:** Jian-bao Yang, Chong-Rui Li, Cheng Wang, Xiao-ping Wei, Tao jing

**Affiliations:** Department of Thoracic Surgery, The Second Hospital & Clinical Medical School, Lanzhou University, Lanzhou, China

**Keywords:** case report, lung cancer, occult adenocarcinoma, pulmonary bullae, spontaneous pneumothorax, video-assisted thoracoscopic surgery (VATS)

## Abstract

**Background:**

Secondary spontaneous pneumothorax (SSP) is a rare but critical initial clinical manifestation of lung cancer, often masquerading as benign bullous disease. The diagnostic challenge is significantly amplified when the underlying malignancy is occult on initial imaging and impalpable during surgical exploration. This report describes a rare case of early-stage adenocarcinoma presenting as SSP in the context of diffuse pulmonary bullae, highlighting the risk of underdiagnosis in high-risk populations.

**Case Description:**

A 55-year-old male with a 60 pack-year smoking history presented with acute-onset right-sided chest pain. Initial high-resolution computed tomography (HRCT) confirmed a right-sided pneumothorax associated with multiple bilateral bullae and localized emphysema; however, no discrete nodules or masses were identified. The patient underwent video-assisted thoracoscopic surgery (VATS) for apical bullae resection and pleurodesis. Intraoperative exploration revealed multiple bullae but no palpable solid lesions. Surprisingly, postoperative histopathological examination identified an infiltrating, moderately differentiated lung adenocarcinoma (6mm in diameter) characterized by 80% acinar and 20% lepidic growth patterns. Immunohistochemical staining was positive for CK7, Napsin A, and TTF-1. Although the patient declined further staging or radical resection, he has remained recurrence-free during 16 months of postoperative follow-up.

**Conclusion:**

Spontaneous pneumothorax in high-risk elderly smokers may mask occult lung cancer, even with negative HRCT findings. Clinicians should maintain high suspicion and routinely utilize intraoperative frozen section analysis for resected bullae to prevent delayed diagnosis and ensure timely oncological management.

## Introduction

Spontaneous pneumothorax (SP) is clinically classified into primary and secondary subtypes, distinguished by the absence or presence of underlying parenchymal disease. Primary spontaneous pneumothorax (PSP) characteristically affects individuals without known pulmonary disorders, with a predilection for young, tall, and slender males. In contrast, secondary spontaneous pneumothorax (SSP) occurs as a complication of pre-existing lung pathologies (1). While the etiologies of SSP are diverse, its occurrence as the sentinel clinical manifestation of primary lung cancer is remarkably infrequent, with an estimated incidence ranging from 0.03% to 0.05% (2). The specific pathophysiological mechanisms through which an early-stage malignancy triggers the rupture of the visceral pleura remain largely elusive (1). This report details the clinical trajectory of a patient presenting with SSP secondary to an occult lung adenocarcinoma, which was initially obscured by the presence of pulmonary bullae and localized emphysematous changes. Through this case, we aim to highlight the diagnostic intricacies and the critical importance of maintaining a high index of oncological suspicion in high-risk patients presenting with atypical pneumothorax.

## Case history/examination

A 55-year-old Chinese male, with a significant tobacco exposure history of 60 pack-years, presented to our thoracic outpatient clinic reporting the acute onset of mild, localized right-sided chest pain of 2 days’ duration. The patient stated that the symptoms emerged spontaneously, without preceding physical exertion or thoracic trauma. Notably, he denied experiencing respiratory distress, chronic cough, hemoptysis, or constitutional symptoms such as fever and unexplained weight loss. His prior medical history was unremarkable, and there was no documented family history of pulmonary or systemic malignancy.

Upon physical examination, the patient was hemodynamically stable and in no acute distress. Thoracic auscultation revealed a unilateral diminution of breath sounds over the right upper lung field, while the contralateral lung demonstrated normal vesicular breath sounds. No palpable superficial lymphadenopathy was identified in the supraclavicular or axillary regions. Initial laboratory evaluations, including a complete blood count (CBC) and a comprehensive metabolic profile, yielded results within physiological reference ranges.

### Diagnostic imaging

Upon acute presentation, initial diagnostic imaging included a frontal chest radiograph (**Figure 1A**), which revealed a right-sided pneumothorax alongside underlying emphysematous changes. Subsequent chest high-resolution computed tomography (HRCT) confirmed the right-sided pneumothorax with approximately 30% pulmonary collapse and demonstrated extensive bilateral pulmonary bullous disease predominantly in the apical segments (**Figure 1B**). Crucially, meticulous radiologic evaluation of the lung parenchyma failed to identify any suspicious solid nodules, sub-solid opacities, or mass-like lesions indicative of malignancy. Based on these findings, a diagnosis of SSP due to bullae rupture was established, without radiographic evidence of an occult neoplasm.

**Figure 1:**
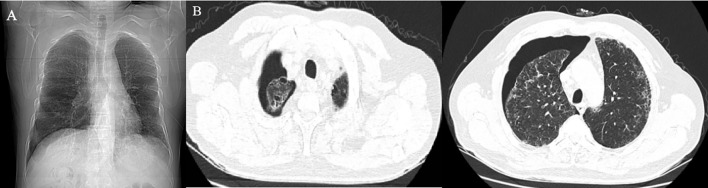
Initial diagnostic imaging upon admission. **(A)** Frontal chest radiograph showing a right-sided pneumothorax and emphysematous changes. **(B)** Axial high-resolution computed tomography (HRCT) slices demonstrating the right-sided pneumothorax and extensive apical bullous disease without evidence of occult malignancy.

### Therapeutic intervention and clinical course

Following a multidisciplinary clinical evaluation, the patient’s condition was consistent with SSP secondary to bullous rupture. Initial management involved the placement of an intercostal chest tube to stabilize the patient and facilitate lung re-expansion. Although the patient exhibited only mild respiratory distress and a moderate volume of pneumothorax, follow-up HRCT performed after lung re-expansion confirmed the presence of prominent, cluster-like apical bullae on HRCT—recognized as a significant risk factor for recurrence—prompted the decision for definitive surgical intervention. Conservative management was deemed insufficient given the high likelihood of future episodes.

After obtaining comprehensive informed consent, the patient underwent video-assisted thoracoscopic surgery (VATS) under general anesthesia. Intraoperative exploration of the right thoracic cavity corroborated the radiographic findings, revealing multiple thin-walled bullae localized in the apical segment of the right upper lobe. A meticulous inspection and palpation of the accessible lung parenchyma were performed; however, no macroscopically suspicious solid nodules, pleural puckering, or indurated lesions were identified. Consequently, a targeted wedge resection of the apical bullae was performed using endoscopic linear staplers, followed by mechanical pleurodesis.

The immediate postoperative course was unremarkable. Following confirmation of complete lung re-expansion and the absence of persistent air leaks, the chest tube was removed on the sixth postoperative day, and the patient was discharged in stable condition. However, the subsequent routine histopathological analysis of the “benign” bullae specimen yielded an incidental and unexpected diagnosis of lung adenocarcinoma. Upon receiving the pathology report, the clinical team strongly recommended further oncological staging—including mediastinal lymph node evaluation and potential radical lobectomy. Due to personal reasons and the absence of symptoms, the patient declined additional invasive interventions, opting instead for a rigorous protocol of serial imaging surveillance.

### Pathological diagnosis

Microscopic examination of the resected specimen from the right upper lobe revealed an infiltrating, moderately differentiated pulmonary adenocarcinoma. The tumor measured 6 mm in its maximum dimension and was characterized by a mixed architectural pattern consisting of 80% acinar and 20% lepidic growth. Cytologically, the malignant cells exhibited moderate nuclear atypia and prominent nucleoli. Despite the proximity to the visceral pleura, no evidence of pleural invasion or lymphovascular space involvement was identified, and the surgical resection margins were histologically clear. To further delineate the lineage, a comprehensive immunohistochemical (IHC) panel was performed. The neoplastic cells exhibited diffuse and strong nuclear positivity for Thyroid Transcription Factor-1 (TTF-1) and Napsin A, along with cytoplasmic staining for Cytokeratin 7 (CK7) (**Figure 2**). These findings confirmed the diagnosis of primary pulmonary adenocarcinoma, staged as pT1aNxMx based on the 8th edition of the AJCC/UICC manual. Following the histopathological diagnosis, an immediate contrast-enhanced CT scan was performed, revealing no evidence of regional lymph node metastasis or distant lesions; the adjacent lung parenchyma showed chronic bullous disease and localized emphysema without additional occult malignant foci.

**Figure 2:**
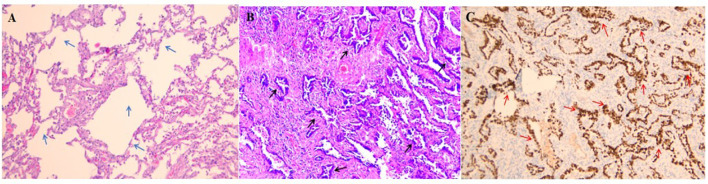
Histopathological and immunohistochemical findings. **(A)** Resected specimen showing pulmonary bullae (blue arrow). **(B)** Moderately differentiated lung adenocarcinoma cells (black arrow) (H&E stain). **(C)** Diffuse nuclear positivity for TTF-1 (red arrow) (IHC stain). Magnification, ×100.

### Follow-up and outcomes

The patient’s postoperative course was uneventful, and a structured oncological follow-up protocol was initiated. An initial follow-up chest CT scan demonstrated complete resolution of the pneumothorax, satisfactory bilateral lung re-expansion, and expected early postoperative changes at the surgical site (**Figure 3A**). A subsequent long-term surveillance chest CT scan performed at 16 months postoperatively confirmed sustained lung re-expansion, stable chronic emphysematous changes, and benign postoperative pleural thickening on the right side (**Figure 3B**). Crucially, neither scan showed any evidence of local tumor recurrence, new suspicious pulmonary nodules, or regional lymphadenopathy. At the latest follow-up, the patient remained completely asymptomatic with excellent clinical recovery.

**Figure 3:**
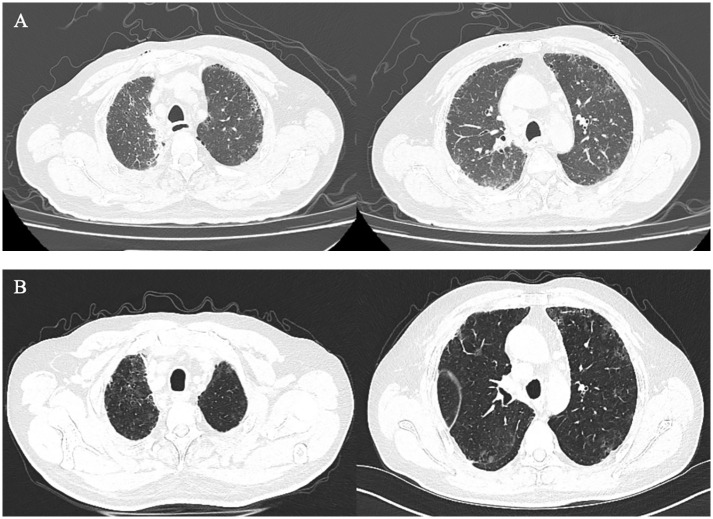
Postoperative follow-up chest CT examinations. **(A)** Short-term postoperative follow-up axial high-resolution computed tomography (HRCT) slice showing complete resolution of the right-sided pneumothorax and satisfactory bilateral lung re-expansion. **(B)** The latest surveillance HRCT slice at 16 months postoperatively demonstrating sustained lung re-expansion and stable chronic emphysematous changes, with no radiological evidence of local tumor recurrence.

## Discussion

The clinicopathological link between SP and underlying primary lung cancer has been documented in the thoracic literature for several decades. Early clinical observations established that a pneumothorax event should not be viewed merely as a simple mechanical failure of the lung tissue, but rather as a potential complication or even the sentinel manifestation of an undiagnosed malignancy (3, 4). While historical evidence often focused on cases involving radiologically apparent or advanced-stage tumors, the present case underscores the continued relevance of this association in the modern era. It demonstrates that even sub-centimeter, occult lesions can trigger pleural compromise, necessitating a high degree of clinical suspicion in high-risk populations regardless of initial imaging findings.

This case report highlights the unusual occurrence of SSP as the initial presentation of lung cancer in a patient with underlying pulmonary bullae and localized emphysema. The association between lung cancer and SP is even more unusual in the absence of a visible mass or nodule on initial imaging, as was the case with our patient. Pneumothorax due to primary lung cancer is rare (5), We searched the PubMed database, identified similar case reports, and performed a retrospective analysis, Based on preoperative screenings, the 14 literature cases showed contrasting clinical pathways (2, 6–14) (**Table 1**). For patients with known preoperative masses (n = 10), malignancy suspicion was integrated into primary planning: 5 achieved simultaneous pneumothorax resolution and radical clearance via single-setting anatomical resection, and 3 utilized frozen sections. Conversely, 3 patients with known masses underwent bullectomy alone without subsequent reoperation, primarily because thorough evaluation revealed advanced-stage disease (e.g., stage IV), rendering the tumors unresectable. These advanced cases were subsequently triaged to non-surgical systemic modalities, such as chemotherapy or targeted therapy, rather than being left untreated. In sharp contrast, for patients without preoperative masses (n = 4), the hidden malignancy was completely omitted from initial strategies due to benign radiological appearances. All 4 underwent simple bullectomy without frozen sections, which elevated their risk of under-treatment and directly resulted in 2 requiring a separate, second radical surgery once final postoperative pathology incidentally disclosed lung cancer, Crucially, we do not suggest two separate operations for patients with radiologically detectable, resectable tumors prior to surgery, who should instead receive single-setting radical resections. Rather, the need for a second repeat surgery applies strictly to highly occult cases resembling our own—where the lack of preoperative radiological suspicion leads to an initial incomplete resection (simple bullectomy), necessitating subsequent secondary radical clearance once the diagnosis is established postoperatively. Overall, smoking and being an elderly male are identified as high-risk factors. In early reported cases, after SP, X-ray was the primary diagnostic tool (3, 15, 16). These patients typically presented with significant air leakage and pulmonary masses, and their lung cancer was relatively advanced at diagnosis. Since the widespread use of chest HRCT scans, most patients with SP first underwent closed thoracic drainage, followed by repeated chest HRCT scans (9, 11). Consequently, early-stage neoplastic lesions were rarely detected in these patients, as the majority of cases had already progressed to an advanced stage by the time of diagnosis. Regrettably, since these procedures were initially planned as elective surgeries for benign pneumothorax, intraoperative frozen section analysis was not routinely performed for incidentally encountered micro-lesions (7, 11, 13), Consequently, the surgical approach focused strictly on the management of the pneumothorax, thereby precluding the immediate implementation of standardized, oncologically sound resections for these unrecognized pulmonary malignancies during the initial operation. The primary reason for this diagnostic omission is that SP remains an exceedingly rare manifestation of early or occult lung cancer, and baseline imaging such as HRCT frequently fails to detect any definitive suspicious masses. Consequently, predicting the underlying malignancy preoperatively is clinically challenging, which underscores the indispensable value of routine postoperative histopathological analysis for all resected bullae specimens, regardless of a benign preoperative appearance. Although repeat surgery could be a potent adjunctive treatment after obtaining final pathology, our literature review identified only two reported cases of similar treatment (2). In these two cases, which were ultimately staged as IA with a maximum tumor diameter of less than 2 cm, repeat surgery was not performed. The primary clinical rationales were that post-operative histopathological evaluation confirmed negative incisional margins, and surveillance HRCT scans after lung re-expansion revealed no definitive residual pulmonary nodules or masses. Furthermore, the patients’ families declined further invasive intervention, opting instead for rigorous follow-up (11). In conclusion, our analysis underscores the clinical value of meticulous intraoperative evaluation for occult malignancy in atypical SP cases. While routine intraoperative frozen section for all SP patients may not be cost-effective, it serves as a critical diagnostic tool for high-risk subgroups, particularly elderly smokers. Furthermore, clinical vigilance should extend to patients with incidental mediastinal lymphadenopathy. As evidenced by previous studies on incidental lung cancer, enlarged lymph nodes—even in the absence of a primary pulmonary mass—warrant careful investigation to exclude an underlying malignant etiology.

**Table 1:** Clinical profiles, surgical interventions, and pathological outcomes of patients segregated by preoperative screening findings (N = 14).

Parameters	Subcategories	With preoperative nodule/mass (n=10)	Without preoperative nodule/mass (n=4)	Total cohort (N = 14)
Demographics				
Gender	Male	9	3	12
	Female	1	1	2
Smoking History	Yes	7	1	8
	No	3	1	4
	Unknown	0	2	2
Clinical Presentation				
Tumor Location	RUL	4	2	6
	RLL	3	0	2
	LUL	3	0	3
	LLL	1	2	3
Management	Preoperative Chest Tube	9	3	12
Intraoperative Diagnosis				
Frozen Section	Performed (Yes)	3	0	3
	Not Performed (No)	7	4	11
Surgical Treatment				
Choice of Surgery	Bullae Resection & Pleurodesis	3	2	5
	Lobectomy or Partial Resection	5	1	6
	Repeat Surgery	0	2	2
	NA	1	0	1
Oncological Outcomes				
Final Pathology	NSCLC	7	3	10
	SCLC	0	1	1
	AAH	1	0	1
	Synovial Sarcoma	1	1	2
Pathological Stage	Stage IA	2	3	5
	Stage IB	1	0	1
	Stage IIA	0	1	1
	Stage IV	3	0	3
	NA	4	0	4

RUL, right upper lobe; RLL, right lower lobe; LUL, left upper lobe; LLL, left lower lobe; NSCLC, non-small cell lung cancer; SCLC, small cell lung cancer; AAH, atypical adenomatous hyperplasia; NA, not available.

The pathophysiology of SSP in patients with lung cancer remains incompletely understood. Possible mechanisms include the rupture of subpleural bullae or emphysematous blebs into the pleural space, direct tumor invasion of the visceral pleura, or tumor-induced airway obstruction leading to a check-valve mechanism and subsequent rupture (16–18). In our patient, the rupture of subpleural bullae was the suspected cause, given the presence of multiple bullae and the absence of visible tumor invasion on imaging.

The diagnosis and treatment of occult lung cancer presenting with SSP is challenging. According to current NCCN guidelines, low-dose spiral HRCT is recommended for early lung cancer screening in elderly patients with a history of long-term smoking (19). Upon inquiry, we learned that the patient had not undergone prior screening due to personal reasons. While early HRCT screening did not detect any suspicious lung cancer signs or overt nodules in this specific patient, from a broader clinical perspective, comparing pre- and post-pneumothorax HRCT images can help clinicians assess subtle structural transformations—such as unexplained localized wall thickening or progressive bullous changes—thereby providing crucial diagnostic clues for occult malignancies.

Preoperative chest HRCT did not reveal distinct lung nodules or masses, and the patient’s SP was attributed to the rupture of pulmonary bullae. Consequently, lung cancer was not initially considered. After specimen removal, no palpable nodules were identified, so a frozen section was not performed. This highlights why intraoperative rapid pathological assessment was not utilized to assist in diagnosing potential malignancy. Failure to consider lung cancer during preoperative or intraoperative assessments may lead to a focus on treating SP while neglecting standardized surgical management of lung cancer, often resulting in the oversight of intraoperative rapid pathology.

Without intraoperative rapid pathology, the final pathological results were available several days postoperatively. As a result, this patient only underwent surgical treatment for pulmonary bullae rather than the appropriate surgery for lung cancer. This likely impacted the cancer’s staging and subsequent decisions regarding adjuvant therapy. Ideally, the patient should have undergone a secondary standardized surgical procedure for lung cancer or at least mediastinal lymph node sampling to clarify the staging. Unfortunately, in this case, the patient and their family declined further invasive examinations or surgical procedures, including mediastinoscopy-guided lymph node biopsy.

This case emphasizes the importance of maintaining a high index of suspicion for lung cancer in patients presenting with SP, especially those with risk factors for malignancy. HRCT of the chest can be helpful in identifying patients with underlying lung diseases associated with occult lung cancer (20, 21), such as bullous emphysema (22). For patients with risk factors for lung cancer, such as a history of smoking and/or elderly, further preoperative examinations may be required, including repeat HRCT examination following closed chest drainage, positron emission tomography (PET)-CT (23, 24) and pathological assessment, even in the absence of visible lung masses. In the diagnostic workup of suspected lung cancer presenting with pneumothorax and atelectasis, we emphasize the importance of closed chest drainage prior to PET-CT scanning to ensure complete lung re-expansion and minimize the risk of false positives due to lung compression (15). In addition, rapid intraoperative pathological diagnosis plays a critical role in reducing the risk of missed diagnoses and subsequent treatment delays (17).

## Limitations

The main limitation of this report is that it is a retrospective review of a single patient case, which limits the generalizability of the study’s findings. Furthermore, the imaging characteristics and biomarkers associated with occult lung cancer that primarily manifests as SP combined with bullous emphysema or emphysematous changes on imaging have not been extensively described, which could provide insights into understanding the tumor biology further. Another significant limitation of this report is the absence of long-term follow-up data for the patient. Currently, the patient has been monitored for 16 months postoperatively without recurrence. The surveillance protocol consisted of contrast-enhanced chest CT scans and serum tumor marker evaluations performed every 6 months during the first year, and scheduled annually thereafter. Given the limited duration of follow-up, we are unable to evaluate the patient’s long-term prognosis, the durability of the surgical intervention, or the potential for lung cancer recurrence or progression.

## Conclusion

This case emphasizes the importance of considering lung cancer in patients presenting with SP, particularly those with risk factors such as smoking and underlying pulmonary diseases.

## Data Availability

The original contributions presented in the study are included in the article/supplementary material. Further inquiries can be directed to the corresponding author.
